# Cholesterol induced-mitochondrial calcium dysregulation facilitates atherosclerosis by promoting lipid accumulation in vascular smooth muscle cells

**DOI:** 10.1186/s43556-025-00384-2

**Published:** 2025-12-02

**Authors:** Zhiwang Zhang, Fan Yang, Wei Wang, Qi Cao, Long Zhang, Yu Zhang, Dong Ma, Xinhua Zhang, Jinkun Wen, Bin Zheng

**Affiliations:** 1 Department of Biochemistry and Molecular Biology, Key Laboratory of Neural and Vascular Biology, Ministry of Education, Hebei Medical University, Shijiazhuang, 050017 China; 2 College of Integrative Medicine, Hebei University of Chinese Medicine, Shijiazhuang, 050200 China; 3 Hebei Key Laboratory of Cardiovascular Homeostasis and Aging, Hebei Medical University, Shijiazhuang, 050017 China

**Keywords:** Atherosclerosis, Mitochondrial calcium, MICU1, MCU inhibitor

## Abstract

Mitochondria play an essential role in regulating various physiological functions including bioenergetics, calcium homeostasis, redox signaling, and lipid metabolism and also are involved in the pathogenesis of cardiovascular diseases. However, the relationship between mitochondrial calcium homeostasis in vascular smooth muscle cells (VSMCs) and atherosclerosis remains poorly understood. Here, we demonstrate that cholesterol induces mitochondrial calcium overload and lipid accumulation in VSMCs, which is resulted from dysregulation of mitochondrial calcium uniporter (MCU), as evidenced by genetic and pharmacologic inhibition of MCU. Furthermore, MCU inhibitors alleviate Western diet-induced atherosclerosis in ApoE-/- mice. Mechanistically, high-fat and high-cholesterol diets induce the contact between mitochondria and the endoplasmic reticulum (ER) in VSMCs as indicated by transmission electron microscopy, proximity ligation assay and immunofluorescence staining, which increases the formation of mitochondria-associated membranes (MAMs), leading to Ca2 + release from the ER into the mitochondria and thus elevating Ca2 + in the mitochondria. Using mitochondrial calcium uptake 1 (MICU1) mutant and Ca2 + detection assay, we confirmed that this increased Ca2 + binds to MICU1, a blocker of MCU, to impair its ability to block MCU, thus enabling the MCU to remain open and resulting in mitochondrial calcium overload. Further, mitochondrial calcium overload dysregulates fatty acid β-oxidation by modulating medium-chain acyl-CoA dehydrogenase (ACADM), thereby leading to lipid deposition. The inhibition of MCU alleviates the pathological changes elecited by cholesterol. Our findings unveil the previously unrecognized role of MAM-MICU1-MCU axis in cholesterol-induced mitochondrial calcium overload and atherosclerosis, indicating that MCU represents a promising therapeutic target for the treatment of atherosclerosis.

**Supplementary Information:**

The online version contains supplementary material available at 10.1186/s43556-025-00384-2.

## Introduction

Atherosclerosis is a chronic inflammatory disease of large and medium-sized arteries, and a leading cause of death and loss of productive life years worldwide. Atherogenesis refers to the development of atheromatous plaques in the inner lining of the arteries [1]. In the initial stage, damage to the endothelium of arteries occurs under the impact of inflammation, lipids, and blood flow [2]. Subsequently, lipoproteins accumulate in the intimal region of the vessel, including low-density lipoproteins (LDLs), especially oxidized LDLs (oxLDL), and remnants of triglyceride-rich lipoproteins [3]. The large accumulation of lipoproteins leads to increased plaque instability, increased risk of fibrous cap rupture, and a sudden increase in the probability of myocardial infarction [4].

Macrophages play a crucial role in plaque formation. Accumulation of cholesterol and other LDL-derived lipids gives rise to lipid-loaded macrophages or foam cells, eventually forming atherosclerotic plaques [5, 6]. However, the importance of VSMCs in the development of atherosclerosis is increasingly on the radar of researchers. Lineage tracing studies have shown that VSMCs can undergo trans-differentiation into macrophage-like and osteochondrogenic descendants [7]. It has also been estimated that in animal models of atherosclerosis, VSMC-derived foam cells could account for as much as 50% of lesional foam cells [8]. Thus, VSMC-derived macrophage-like cells and foam cells have been considered to be an important contributor to atherosclerosis and necrotic core formation in plaques. Despite these advances in understanding the mechanisms of atherogenesis in the past decades, it remains poorly understood how lipid accumulation is regulated in VSMCs.

Mitochondria integrate information from various intracellular compartments to regulate cellular signaling and metabolism and may respond to various extracellular signaling events [9], thus exerting a critical role in regulating various physiological and pathological processes. Mitochondrial dysfunction contributes to the pathologies of cardiovascular disorders [10]. Our recent study confirmed that mitochondrial calcium homeostasis imbalance precedes lipid metabolism disorders and regulates hepatic lipid deposition [11]. However, the link between mitochondrial calcium homeostasis imbalance and lipid deposition in VSMCs remains to be investigated.

Mitochondrial Ca^2^⁺ uptake is accomplished by the mitochondrial calcium uniporter (MCU) located in the inner mitochondrial membrane (IMM) [12]. The mitochondrial calcium uniporter is a complex composed of multiple subunits, consisting of at least four components: the ion-conducting channel (MCU) [13], the essential transmembrane subunit (EMRE), and the mitochondrial inner membrane outer gate proteins (MICU1 and MICU2) [14]. The homologous tetramer of MCU forms the MCU channel. EMRE, like a hinge, connects MCU with mitochondrial calcium uptake 1 (MICU1). MICU1 plays an inhibitory role on the channel and acts as a control switch of the MCU channel, demonstrating that the interaction of MCU, EMRE and MICU1/2 plays an important role in the regulation of MCU channel activity.

In this study, we made an original discovery that a high-fat, high-cholesterol diet promotes mitochondrial calcium overload in VSMCs, and systematically revealed the mechanism of mitochondrial calcium overload during atherosclerosis. We also confirmed that mitochondrial calcium overload may regulate lipid metabolism by modulating fatty acid β-oxidation through ACADM. Finally, we found that MCU inhibitors alleviate lipid deposition and atherosclerosis induced by a high-fat, high-cholesterol diet both in vivo and in vitro. These findings further elucidate the pathogenesis of atherosclerosis and provide direction for the development of therapeutic approaches.

## Results

### Cholesterol induced lipid accumulation and mitochondrial calcium overload in VSMCs

**Fig. 1:**
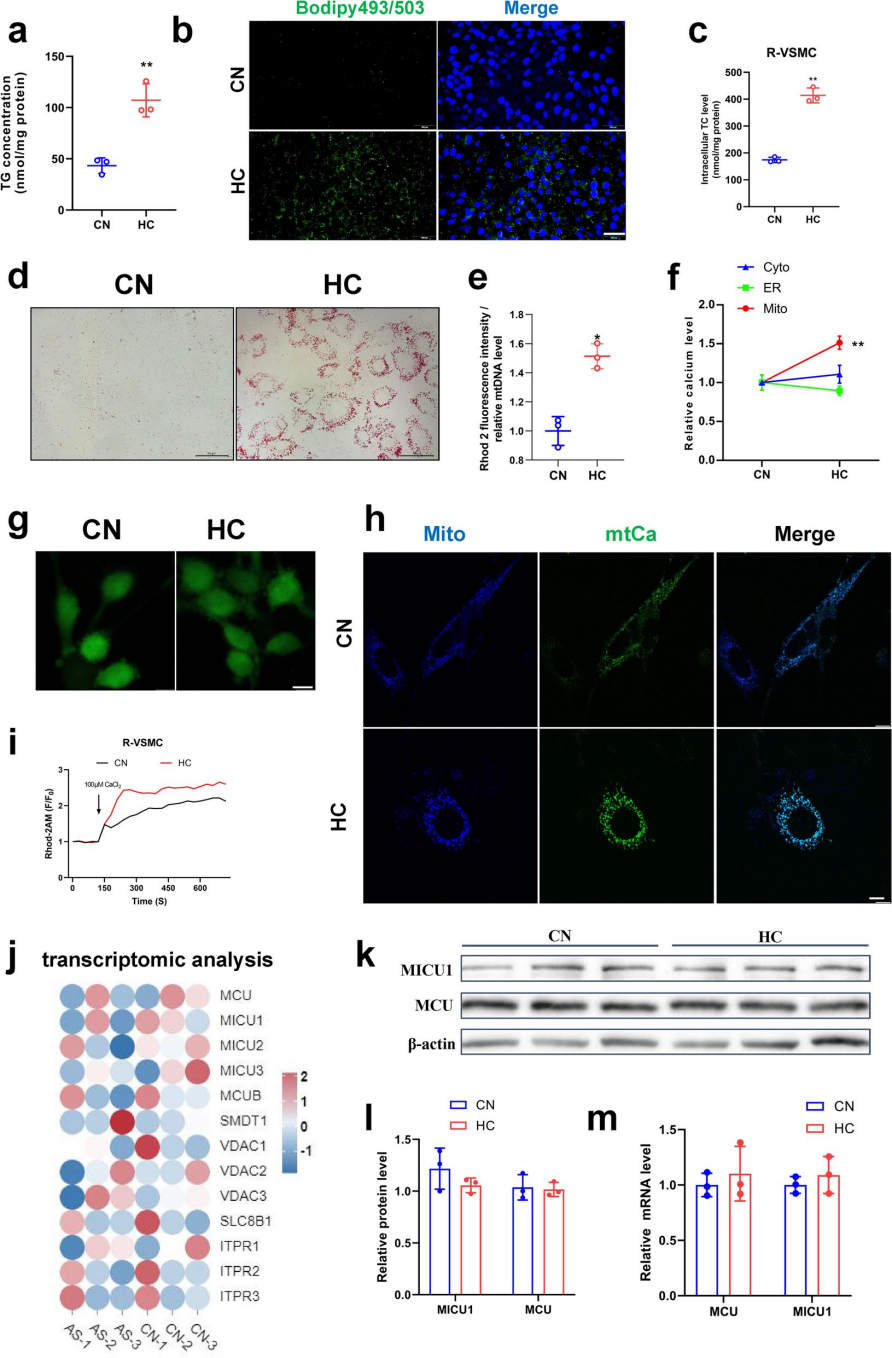
The effect of cholesterol on mitochondrial calcium homeostasis and lipid deposition in smooth muscle cells. **a** Triglyceride content. **b** Bodipy staining of lipid droplets bar = 200 μm. **c** Total cholesterol content (Rat-VSMC). **d** Oil red O staining of lipid droplets (Rat-VSMC) bar = 50 μm. **e** Mitochondrial calcium level (mitochondrial calcium staining fluorescence intensity/mitochondrial DNA quantity). **f** Calcium changes after cholesterol addition in cytoplasm, ER, and mitochondria (pCMV-CEPIA2mt). **g** Calcium staining of cytoplasm (Fluo-4AM) bar = 10 μm. **h** Calcium changes after cholesterol addition. Mitochondrial staining (mito-BFP), mitochondrial calcium fluorescence (pCMV-CEPIA2mt) bar = 10 μm. **i** Calcium changes after cholesterol addition. Mitochondrial calcium change curve(F/F_0_), CaCl_2_ to stimulate mitochondrial calcium uptake. **j** Heat map of mitochondrial calcium related genes. AS mean patients with atherosclerosis. The human data of atherosclerosis were taken from the Gene Expression Omnibus, Accession Number GSE226790. **k**-**l** MCU, MICU1 protein expression level. (**m**) MCU, MICU1 mRNA expression level

Hypercholesterolemia is the major risk factor for atherosclerotic plaque formation. We found that 30 mg/mL cholesterol addition upregulated triglyceride and cholesterol accumulation by more than twofold in VSMCs (Fig. 1a, c&S1a, b). Staining of lipid droplets by oil red O and bodipy similarly confirmed that cholesterol promoted the accumulation of lipid droplets (Fig. 1b, d&S1c, d). Simultaneously, we examined Ca^2+^ levels in the endoplasmic reticulum (ER), cytoplasm, and mitochondria and found that calcium levels elevated specifically in the mitochondria (Fig. 1e), while high cholesterol had no significant effect on Ca^2+^ levels in the cytoplasm and ER (Fig. 1f&g). To corroborate these findings, Ca^2+^ in mitochondria was assayed using Rhod-2AM, pCMV-CEPIA2mt fluorescent vectors which is specifically expressed in mitochondria and produces green fluorescence when bound to Ca^2+^ [15], and the results demonstrated a significant increase in mitochondrial calcium levels following cholesterol treatment (Fig. 1h, i&S1e). However, the expression of mitochondrial calcium channel-related genes (MCU, MICU1, MICU2, MICU3, MCUB, SMDT1, VDAC1, VDAC2, VDAC3, SLC8B1, ITPR1, ITPR2 ITPR3) had no significant differences between normal and patients with atherosclerosis based on transcriptomic data from a database (Fig. 1j). Additionally, Western blot and mRNA expression assays for the mitochondrial calcium channel proteins MCU and MICU1 also had no significant differences (Fig. 1k-m). These results suggest that cholesterol-induced mitochondrial calcium overload in VSMCs may not occur through the regulation of mitochondrial calcium channel protein expression, but rather through the modulation of their channel activity.

### Inhibition of mitochondrial calcium overload alleviated cholesterol-induced lipid deposition in VSMCs by regulating glycerophospholipid metabolism

**Fig. 2:**
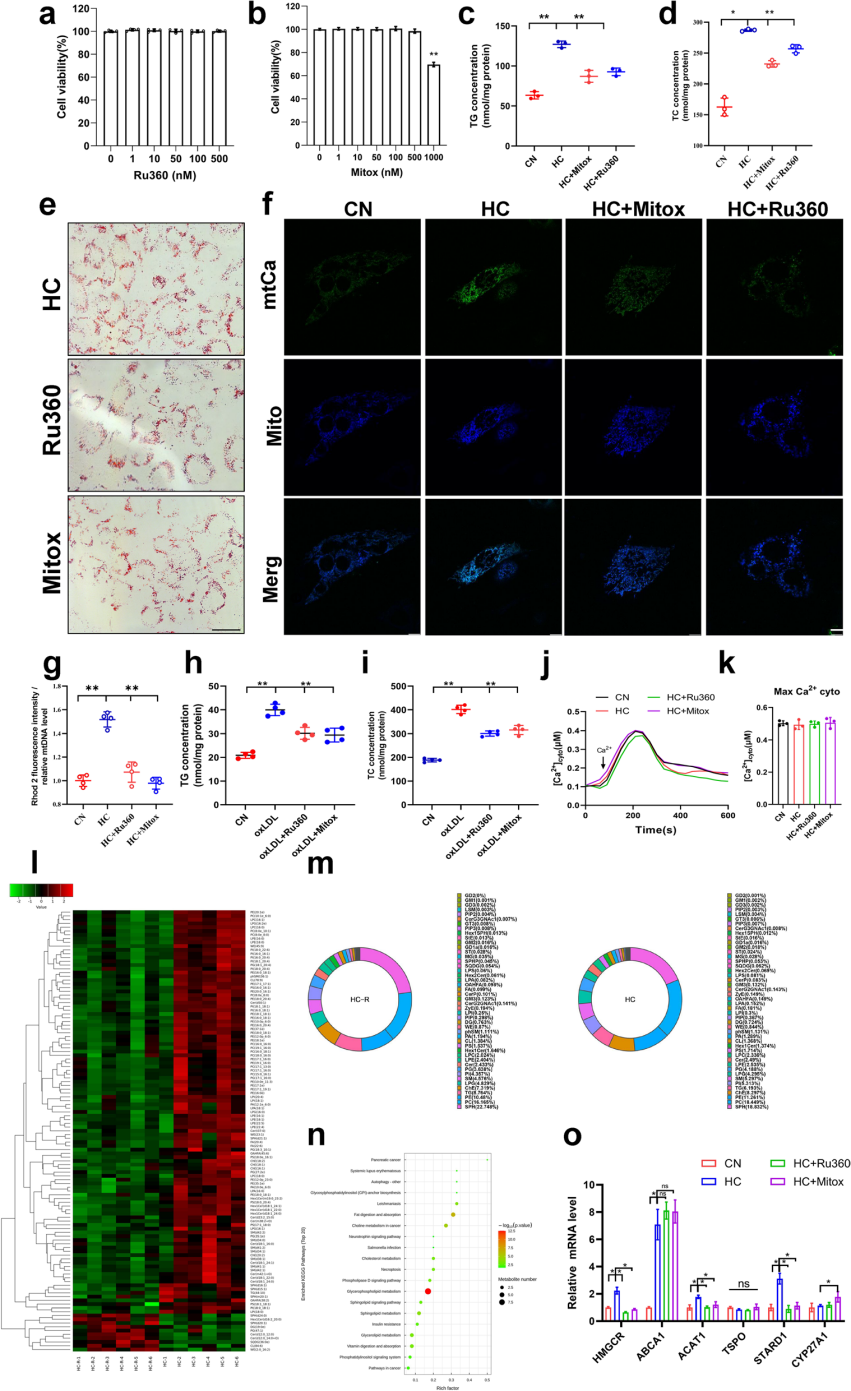
MCU inhibitors regulate mitochondrial calcium homeostasis and lipid deposition. **a**, **b** CCK-8 Cell viability after MCU inhibitor treatment 24 h. **c** Triglyceride content. CN mean control, HC mean cholesterol addition, HC + Ru360 mean cholesterol and Ru360 addition, HC + Mitox mean cholesterol and mitoxantrone addition. **d** Total cholesterol content. **e** Oil red O staining of lipid droplets bar = 10 μm. **f** Calcium changes after cholesterol addition and MCU inhibitor treatment. Mitochondrial staining (mito-BFP), mitochondrial calcium fluorescence (pCMV-CEPIA2mt) bar = 10 μm. **g** Mitochondrial calcium level. **h** Triglyceride content. **i** Total cholesterol content. **j** Mean time courses from SOCE-associated cyto [Ca^2+^] (fura2). **k** Maximum cyto [Ca.^2+^]. **l** Metabolite expression heatmap in lipidomics after cholesterol addition and MCU inhibitor treatment. **m** Proportion of metabolite types. **n** KEGG Enrichment Analysis. **o** mRNA expression levels of cholesterol metabolism related genes. *Hmgcr* (3-hydroxy-3-methylglutaryl-Coenzyme A reductase), *Abca1* (ATP-binding cassette transporter A1), *Acat1* (Acetyl-Coenzyme A acetyltransferase 1), *Tspo* (Translocator Protein), *Stard1* (Steroidogenic Acute Regulatory Protein 1), *Cyp27a1* (cytochrome P450, family 27, subfamily A, polypeptide 1)

In order to detect whether cholesterol affected mitochondrial calcium homeostasis, two inhibitors of MCU channels, Ru360 and mitoxantrone were used to block MCU channels in VSMCs. CCK-8 experiments confirmed that 0–500nM concentrations of these two inhibitors had no effect on VSMC viability (Fig. 2a&b). However, both inhibitors (10 nM) significantly reduced cellular cholesterol content and the formation of lipid droplets (Fig. 2c-e), and reduced cholesterol-induced mitochondrial calcium overload (Fig. 2f, g&S1f). Another MCU inhibitor, Ru265, also alleviated cholesterol-induced lipid deposition (Fig. S1g&h). In oxLDL-induced atherosclerosis cellular models, the same results were observed: oxLDL induced mitochondrial calcium overload and lipid deposition, both of which were significantly alleviated by the two inhibitors (Fig. 2h&i). These results demonstrated that inhibition of mitochondrial calcium overload alleviated cholesterol-induced lipid deposition. Absolute quantification of cytoplasmic calcium revealed no significant effect of cholesterol on its levels, neither inhibitor affected cytoplasmic calcium, confirming that both agents specifically inhibit MCU activity (Fig. 2j&k). Untargeted lipidomics data confirmed that inhibition of mitochondrial calcium significantly reduced the deposition of the vast majority of lipid species in VSMCs (Fig. 2l). Interestingly, MCU inhibitors had no significant effect on the percentage of lipid species, suggesting that lowering mitochondrial calcium may modulate lipid deposition through an overall reduction in lipid content (Fig. 2m). KEGG pathway enrichment analysis showed that the major differential pathways were focused on fat digestion and absorption, glycerophospholipid metabolism, and other pathways (Fig. 2n). Validation of the above results at the mRNA expression level confirmed that Ru360 and mitoxantrone reduced the mRNA levels of *HMGCR*, *ACAT1* and *STARD1* (Fig. 2o), three of which regulate, respectively, cholesterol synthesis, esterification, and metabolism in the mitochondria.

### Mitochondrial calcium overload may affect lipid deposition by regulating ACADM expression and oxygen consumption rate

**Fig. 3:**
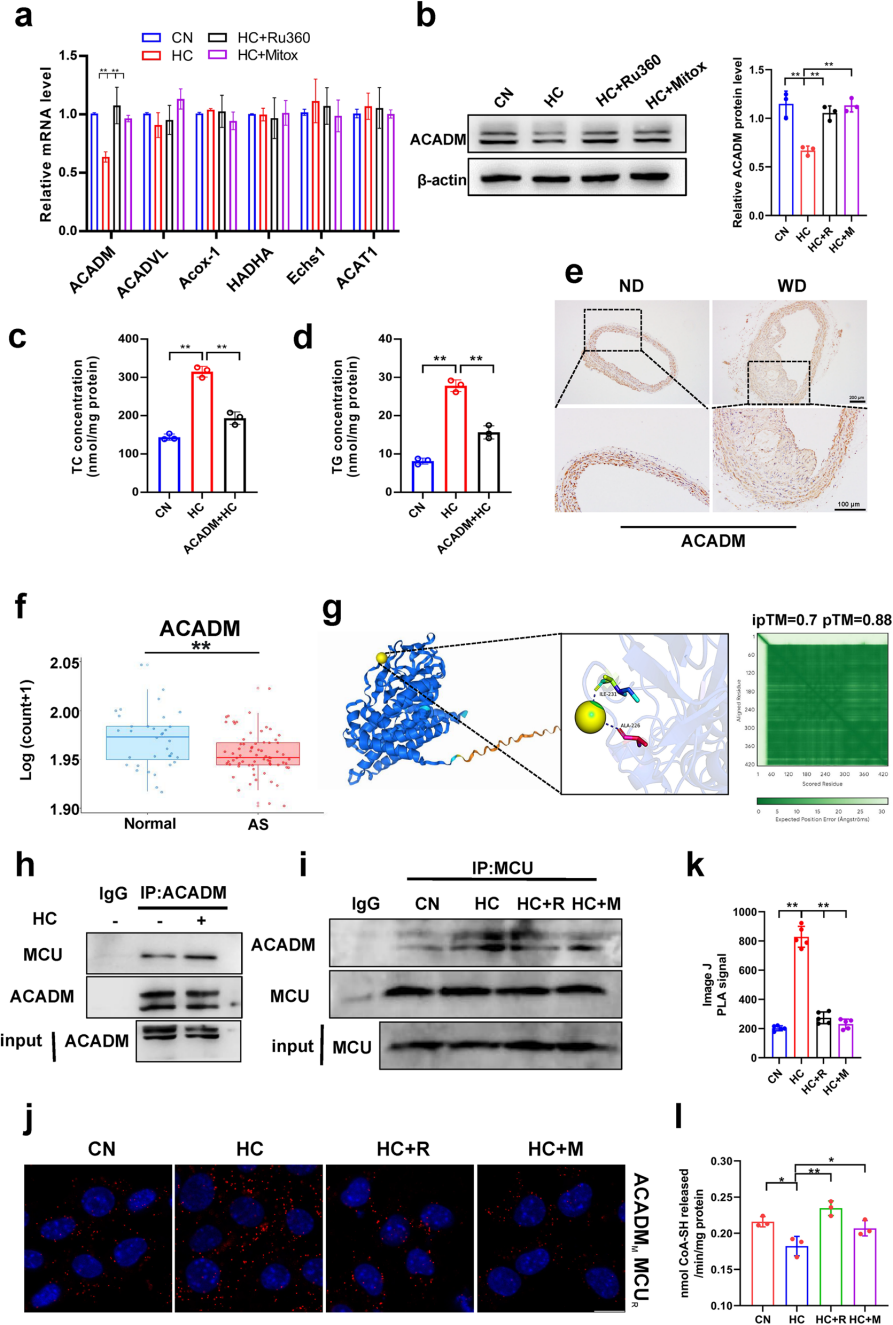
Mitochondrial calcium regulates ACADM expression and oxygen consumption rate. **a** mRNA expression levels of fatty acid β-oxidation genes. *Acadm* (Medium-chain acyl-CoA dehydrogenase), *Acadvl* (Very long chain acyl-coA dehydrogenase deficiency) *Acox-1* (acyl-CoA oxidase), *Hadha,* (hydroxyacyl-CoA dehydrogenase trifunctional multienzyme complex subunit alpha). *Echs1* (enoyl-CoA hydratase 1), *ACAT1* (acyl-CoA: cholesterol acyltransferase 1). CN mean control, HC mean cholesterol addition, HC + R mean cholesterol and Ru360 addition, HC + M mean cholesterol and mitoxantrone addition. **b** ACADM protein expression level. **c** Total cholesterol content. CN mean pCMV; HC mean pCMV + cholesterol; ACADM + HC mean ACADM + cholesterol. **d** Triglyceride content. CN mean pCMV; HC mean pCMV + cholesterol; ACADM + HC mean ACADM + cholesterol. **e** ACAMD immunohistochemical staining. ND mean normal diet, WD mean west diet. bar = 100 μm (**f**) *Acadm* mRNA expression level (GSE100927). **g** Alphafold3 predicts Ca^2+^ binding sites to ACADM. **h** Co-Immunoprecipitation, IP: ACADM, IB: MCU. **i** Co-Immunoprecipitation, IP: MCU, IB: ACADM. **j** Proximity ligation assay, antibody ACADM (mouse); antibody MCU (rabbit). **k** Image J PLA signal. (**l**) CPT1 enzymatic activity

The above studies confirm that mitochondrial calcium regulates lipid deposition in smooth muscle cells, but the exact regulatory mechanism is not clear. Here, we examined genes for fatty acid β-oxidation in mitochondria using qPCR and found that cholesterol reduced ACADM expression and that MCU inhibitors mitigated its induced reduction (Fig. 3a). Western blot also confirmed this result (Fig. 3b). Subsequently, we expressed the ACADM vector into smooth muscle cells and found that it could alleviate cholesterol-induced lipid deposition (Fig. 3c&d). Immunohistochemical staining of mouse aorta and human datasets from public databases also showed that ACADM expression was significantly reduced in atherosclerosis (Fig. 3e, f&S2a). UniProt was used to query the amino acid sequence of ACADM, and alphafold3 was used to predict the binding status of calcium and ACADM. The weighted combination of pTM and ipTM was used as the model confidence measure, and the larger the value, the higher the confidence, and the results of molecular docking modeling showed that there was a strong binding site for calcium and ACADM (Fig. 3g). In addition, immunoprecipitation results revealed that cholesterol enhances the binding between ACADM and MCU (Fig. 3h&i). MCU inhibitors reduce cholesterol-induced interaction between MCU and ACADM (Fig. 3i). Proximity ligation assay (PLA) results indicate that cholesterol treatment increases the connection between ACADM and MCU (Fig. 3j&k), confirming that cholesterol promotes interaction between ACADM and MCU, while inhibitors reduce their interaction. Cholesterol regulation of ACADM may influence mitochondrial fatty acid oxidation. Therefore, we assessed CPT1 activity as an indicator of cellular fatty acid oxidation levels. Results revealed that cholesterol reduced fatty acid oxidation levels, while the inhibitor alleviated the cholesterol-induced decrease in fatty acid oxidation (Fig. 3l). This suggests that cholesterol may regulate fatty acid oxidation via ACADM by modulating mitochondrial calcium.

ATP content measurements also confirmed this conjecture, with cholesterol lowering ATP levels and MCU inhibitors rebounding ATP levels (Fig. S2b). Mitochondrial calcium homeostasis is critical for mitochondrial function. For further study, we tested mitochondrial stress using Seahorse XF analyser (Fig. S2c). The results likewise confirmed that cholesterol decreased basal respiration rate, maximal respiration rate, proton leakage respiration, and ATP production (Fig. S2d-g). Whereas neither cholesterol nor MCU inhibitors had a significant effect on the coupling efficiency of respiration (Fig. S2h). These results suggest that mitochondrial calcium homeostasis might influence lipid deposition by regulating mitochondrial function. Mitochondrial function might be impaired by oxidative stress. Our detection of reactive oxygen species (ROS), one of the oxidative stress markers, suggests that mitochondrial calcium accumulation can impair mitochondrial function by ROS-induced mitochondrial oxidative stress (Fig. S2i).

### Ru360 and mitoxantrone alleviated Western diet-induced atherosclerosis in ApoE^−/−^ mice

**Fig. 4:**
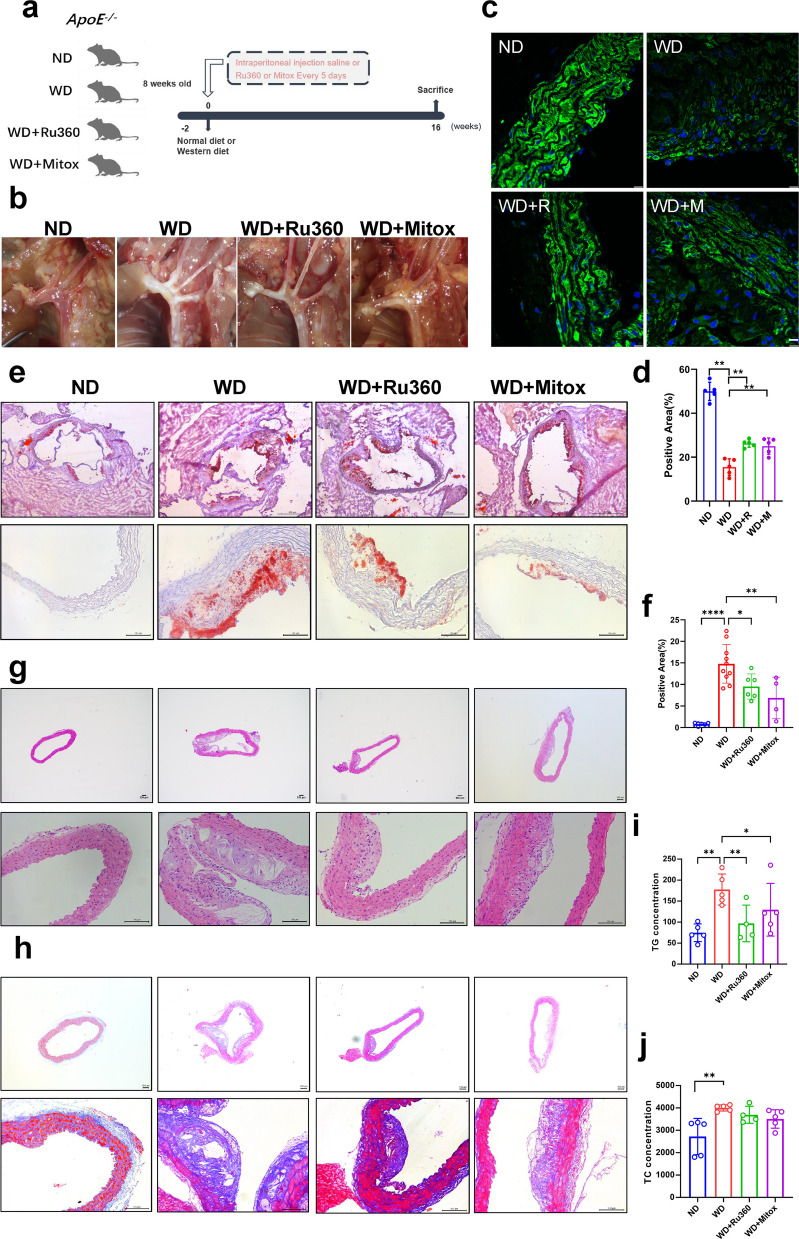
MCU inhibitors alleviates atherosclerosis induced by WD. **a** Experimental plan graphical abstract. CN, normal diet with injected saline; WD, western diet with injected saline; WD + Ru360, western diet with injected Ru360; WD + Mitox, western diet with injected mitoxantrone. **b** Aortic arch photo. **c** α-SMA immunofluorescence staining bar = 10 μm. **d** Immunofluorescence staining positive area. **e** Oil red O staining bar = 100 μm. **f** Proportion of Oil Red O staining positive areas. **g** HE staining bar = 100 μm. **h** Masson staining bar = 100 μm. **i** Serum triglyceride content. **j** Serum Total cholesterol content

The above findings suggest that MCU inhibitors could alleviate lipid deposition in VSMCs. Therefore, we investigated the therapeutic effects of two MCU inhibitors in an animal model. We used ApoE^−/−^ mice and fed them either a normal diet (ND) or a Western diet (WD), while administering intraperitoneal injections of saline or MCU inhibitors (Fig. 4a). The aortas of mice were collected after 16 weeks of feeding and photographed. The photo of the aortic arch shows that the Western diet induced the production of a large number of plaques, proving the successful construction of the model. As expected, Ru360 and mitoxantrone significantly reduced plaque deposition in the aortic arch (Fig. 4b). α-SMA immunofluorescence staining confirmed that the VSMCs markers in the WD group were significantly reduced, while treatment with MCU inhibitors led to a significant recovery (Fig. 4c&d). Aortic oil red O staining and statistics showed that lipid droplet accumulation was significantly increased in the WD mice, whereas both MCU inhibitors alleviated lipid droplet accumulation (Fig. 4e&f). Similarly, HE staining showed that WD induced significant increase in plaque formation in the aorta of mice, and MCU inhibitors alleviated WD induced plaque formation (Fig. 4g). Masson staining of collagen fibers confirmed that WD induced massive collagen deposition in the aorta, which was alleviated by MCU inhibitors (Fig. 4h). In addition, measurements of serum triglycerides and total cholesterol showed that WD significantly increased serum lipid levels, which were attenuated by both inhibitors (Fig. 4i&j). The above results demonstrated that MCU inhibitors could alleviate the atherosclerotic process induced by WD in ApoE^−/−^ mice.

### Mitochondria-associated membranes regulated mitochondrial calcium homeostasis and influenced lipid deposition in VSMCs

**Fig. 5:**
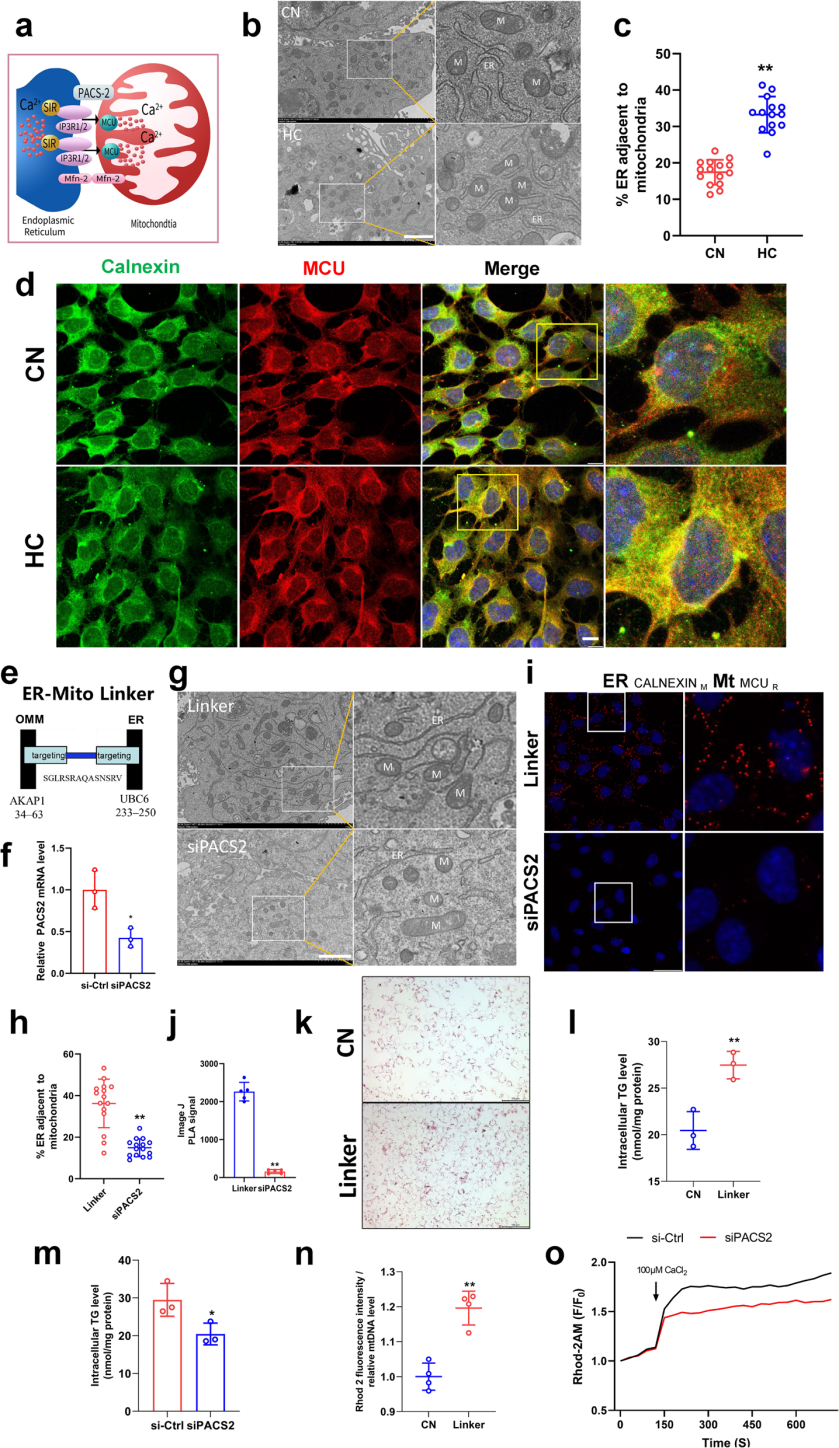
MAMs regulate mitochondrial calcium homeostasis and lipid deposition. **a** MAM schematic. **b** Transmission electron microscopy bar = 2 μm. **c** MAM quantification. **d** Calnexin (green) and MCU (red) immunofluorescence staining bar = 10 μm. **e** ER-Mito linker schematic. **f** *pacs2* mRNA expression levels. **g** Transmission electron microscopy bar = 2 μm. Linker mean ER-Mito linker, siPACS2 mean siRNA for PACS2. **h** MAM quantification. **i** Proximity ligation assay, antibody Calnexin (mouse); antibody MCU (rabbit). **j** Image J PLA signal. **k** Oil red O staining of lipid droplets bar = 100 μm. **l** Triglyceride content. (m) Triglyceride content. **n** Mitochondrial calcium level. **o** Mitochondrial calcium change curve(F/F_0_)

The studies mentioned above confirm that cholesterol induced mitochondrial calcium overload, and that blocking the mitochondrial calcium uptake by inhibiting MCU could alleviate lipid deposition by enhancing mitochondrial function. However, the mechanism by which cholesterol induces mitochondrial calcium overload remains unclear. The mitochondria-associated membrane (MAM), also known as the mitochondria-associated endoplasmic reticulum membrane, comprises a region of the endoplasmic reticulum rich in multiple lipid biosynthetic enzymes. This region reversibly connects to mitochondria and plays a pivotal role in cellular calcium homeostasis, particularly mitochondrial calcium homeostasis (Fig. 5a). Ultrastructural analysis by transmission electron microscopy found that cholesterol significantly increased ER-mitochondrial contacts (Fig. 5b&c), indicating that mitochondrial calcium overload might originate from increased formation of MAM. Further, immunofluorescence staining of the ER protein calnexin and the mitochondrial protein MCU was performed, and showed that cholesterol similarly increased the co-localization of mitochondria and ER (Fig. 5d). PLA results also confirmed increased contact between mitochondria and the ER (Fig. S3a&b). To further investigate the effect of MAM on mitochondrial calcium and lipid accumulation, we designed the ER-Mito linker vector [11] that connects ER and mitochondria to simulate the formation of MAM (Fig. 5e). At the same time, we inhibited the expression of connexin Phosphofurin Acidic Cluster Sorting protein 2 (PACS2) in MAM by specific siRNA (Fig. 5f). Transmission electron microscopy revealed that ER-Mito linker increased the interconnection between ER and mitochondria, while inhibiting PACS2 decreased their contacts, which was consistent with our expectations (Fig. 5g&h). PLA results similarly confirmed that the number of contact sites between mitochondria and the endoplasmic reticulum in the Linker group exceeded that in the siPACS2 group by more than tenfold (Fig. 5i&j). Moreover, Linker increased cellular lipid accumulation (Fig. 5k, l&S3c, d), while siPACS2 decreased lipid accumulation (Fig. 5m). Assays of mitochondrial calcium levels showed that increasing contact between the endoplasmic reticulum and mitochondria by ER-Mito linker elevated mitochondrial calcium (Fig. 5n) and inhibiting their contacts decreased mitochondrial calcium levels (Fig. 5o). The above results demonstrated that increased MAM in VSMCs leads to mitochondrial calcium overload and promotes lipid deposition.

### Mitochondrial calcium uniporter is critical in mitochondrial calcium homeostasis and lipid accumulation

**Fig. 6:**
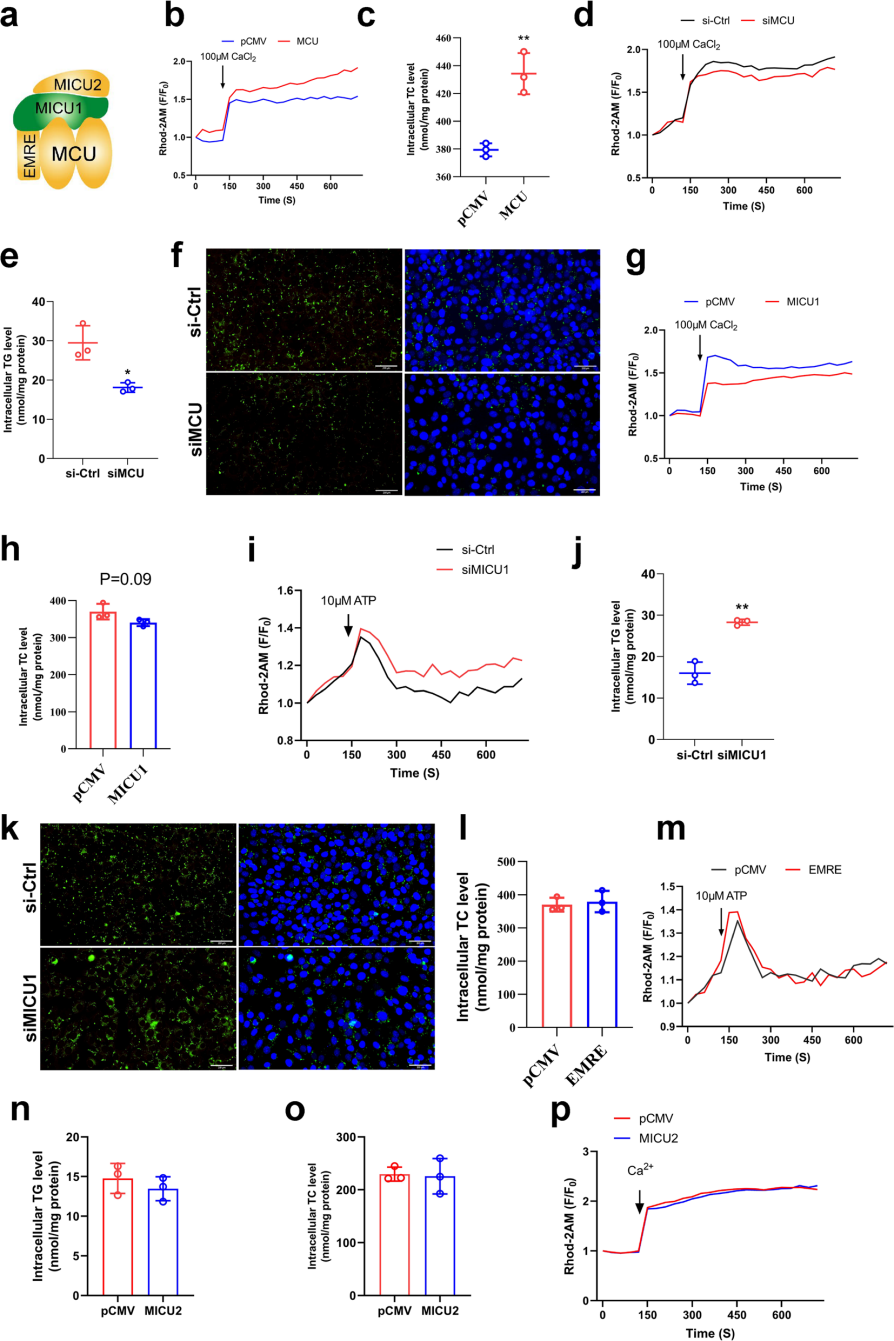
MCU regulates mitochondrial calcium homeostasis and lipid accumulation. **a** MCUs channel (Closed status). **b** Mitochondrial calcium change curve(F/F_0_) after overexpression MCU. CaCl_2_ to stimulate mitochondrial calcium uptake. **c** Total cholesterol content. **d** Mitochondrial calcium change curve(F/F_0_) after siMCU. **e** Triglyceride content. **f** Bodipy staining of lipid droplets bar = 200 μm. **g** Mitochondrial calcium change curve(F/F_0_) after overexpression MICU1. **h** Total cholesterol content. **i** Mitochondrial calcium change curve(F/F_0_) after siMICU1. ATP to stimulate mitochondrial calcium uptake. **j** Triglyceride content. **k** Bodipy staining of lipid droplets bar = 200 μm. **l** Total cholesterol content. **m** Mitochondrial calcium change curve(F/F_0_) after overexpression EMRE. **n** Triglyceride content. **o** Total cholesterol content. **p** Mitochondrial calcium change curve(F/F_0_) after overexpression MICU2

Mitochondrial calcium homeostasis is regulated by the MCU complex consist of EMRE, MICU1, MICU2 (Fig. 6a). First, we overexpressed MCU in VSMCs (Fig. S4a) and found that MCU overexpression promoted mitochondrial calcium overload (Fig. 6b&S4f) while increased lipid accumulation (Fig. 6c&S4b). Subsequently, we found that inhibition of MCU expression by specific siRNA (Fig. S4c) could reduce mitochondrial calcium levels (Fig. 6d&S4g) and alleviated lipid accumulation (Fig. 6e&f). This indicates that the MCU is crucial for mitochondrial calcium homeostasis and lipid deposition, consistent with expectations since the MCU serves as the primary channel for mitochondrial calcium uptake. It also demonstrates that mitochondrial calcium homeostasis dysregulation promotes lipid deposition. Furthermore, we overexpressed MICU1 (Fig. S4d) and found that overexpression of MICU1 reduced mitochondrial calcium (Fig. 6g&S4h) but had no effect on lipid deposition (Fig. 6h). Interestingly, inhibition of MICU1 expression by specific siRNA (Fig. S4e) increased mitochondrial calcium (Fig. 6i) and increased triglyceride accumulation (Fig. 6j&k). The overexpression results of EMRE and MICU2 in VSMCs showed that both of them had no significant effects on mitochondrial calcium and TCs accumulation (Fig. 6l-p). The results indicated that MCU is essential for maintaining mitochondrial calcium homeostasis and regulating lipid deposition in VSMCs. Notably, while the overexpression of MICU1 inhibited mitochondrial calcium elevation but had no effect on lipid deposition, this phenomenon warrants further consideration.

### Cholesterol invalidated the function of mitochondrial calcium uptake 1 and led to persistent open of mitochondrial calcium uniporter

**Fig. 7:**
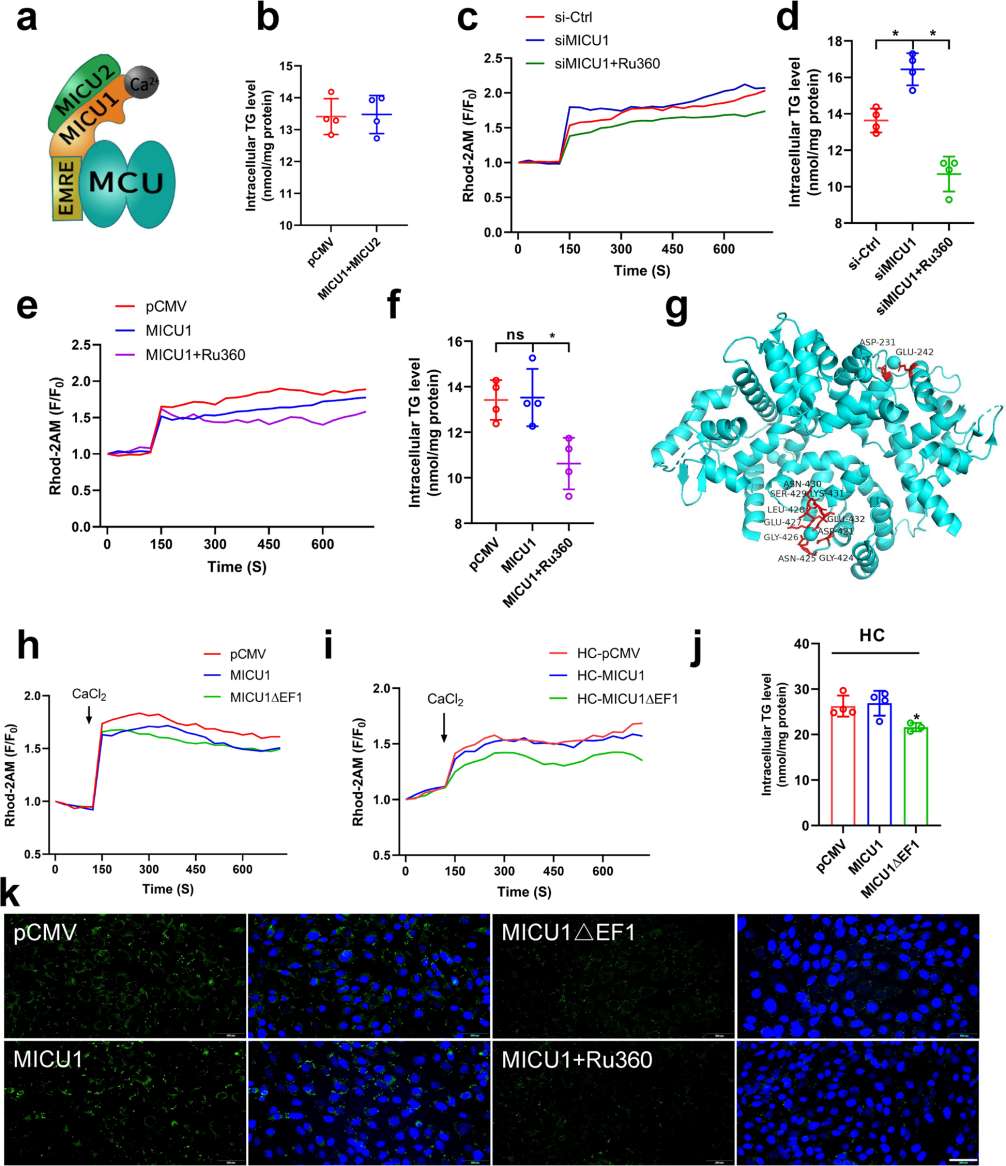
Calcium ions bind to MICU1 to keep the MCU open (**a**) MCUs channel (Open status). **b** Triglyceride content. **c** Mitochondrial calcium change curve(F/F_0_), si-Ctrl mean control, siMICU1 mean siRNA for MICU1, siMICU1 + Ru360 mean siRNA for MICU1 and Ru360 addition. **d** Triglyceride content. **e** Mitochondrial calcium change curve(F/F_0_), pCMV mean control, MICU1 mean overexpression MICU1, MICU1 + Ru360 mean overexpression MICU1 and Ru360 addition. **f** Triglyceride content. **g** MICU1 mutant (MICU1ΔEF1) schematic. **h**, **i** Mitochondrial calcium change curve(F/F_0_). pCMV mean control, MICU1 mean overexpression MICU1, MICU1ΔEF1 mean MICU1 mutant, HC mean cholesterol addition. **j** Triglyceride content. **k** Bodipy staining of lipid droplets bar = 200 μm

The above results reveal that MICU1 regulates mitochondrial calcium and lipid deposition under certain conditions, but the underlying mechanisms require further investigation. Research indicates that MICU2 can act synergistically with MICU1 to co-regulate mitochondrial calcium-control homeostasis (Fig. 7a). Therefore, we overexpressed both MICU1 and MICU2 in VSMCs and found that their overexpression had no effect on lipid accumulation (Fig. 7b), it indicates that MICU2 may not be involved in the regulation of micu1 on MCU. Further studies revealed that siMICU1 elevated mitochondrial calcium levels, and Ru360 alleviated the siMICU1-induced elevation of mitochondrial calcium (Fig. 7c), as well as its induced increase in lipid accumulation (Fig. 7d). Surprisingly, overexpression of MICU1 reduced mitochondrial calcium but had no effect on TG accumulation, and Ru360 further reduced its mitochondrial calcium and decreased lipid deposition (Fig. 7e&f). The above results revealed that overexpression of MICU1 reduced mitochondrial calcium but had no effect on TGs accumulation, which was contrary to our expectations.

The discrepancies between the expected results and the actual results prompted us to consider whether experimental conditions used in calcium and lipid assay contributed to these discrepancies. In our experiments, the mitochondrial calcium assay was performed under conditions without cholesterol addition, whereas the TGs assay was performed under conditions with cholesterol addition in order to promote lipid deposition. Given that cholesterol was the only variable factor, we hypothesize that cholesterol was responsible for the ineffectiveness of MICU1 in reducing mitochondrial calcium levels.

The above study has confirmed that cholesterol induces MAM formation (Fig. 5), which transported calcium into mitochondria, so it is possible that excessive calcium associates with MICU1 to prevent its interaction with MCU, thus shutting down the MCU. To verify our hypothesis, we mutated EF1 (Fig. 7g), the chiral region of MICU1 that senses Ca^2+^ concentration, to reduce its sensitivity to calcium ions. We found that both EF1-mutated MICU1 and wild-type MICU1 reduced mitochondrial calcium (Fig. 7h). However, wild-type MICU1 could not reduce mitochondrial calcium after the addition of cholesterol, whereas EF1-mutated MICU1 could still decrease mitochondrial calcium in the presence of cholesterol (Fig. 7i). Further, TG assay and lipid droplet staining also confirmed that wild-type MICU1 had no effect on lipid accumulation in VSMCs, whereas EF1-mutated MICU1 could alleviate cholesterol-induced lipid deposition (Fig. 7j&k). These results indicate that cholesterol abolishes the ability of wild-type MICU1 to reduce mitochondrial calcium levels, whereas MICU1 mutants can still reduce mitochondrial calcium in the presence of cholesterol. This demonstrates that cholesterol inactivates MICU1 by occupying its chiral region with calcium ions.

**Fig. 8:**
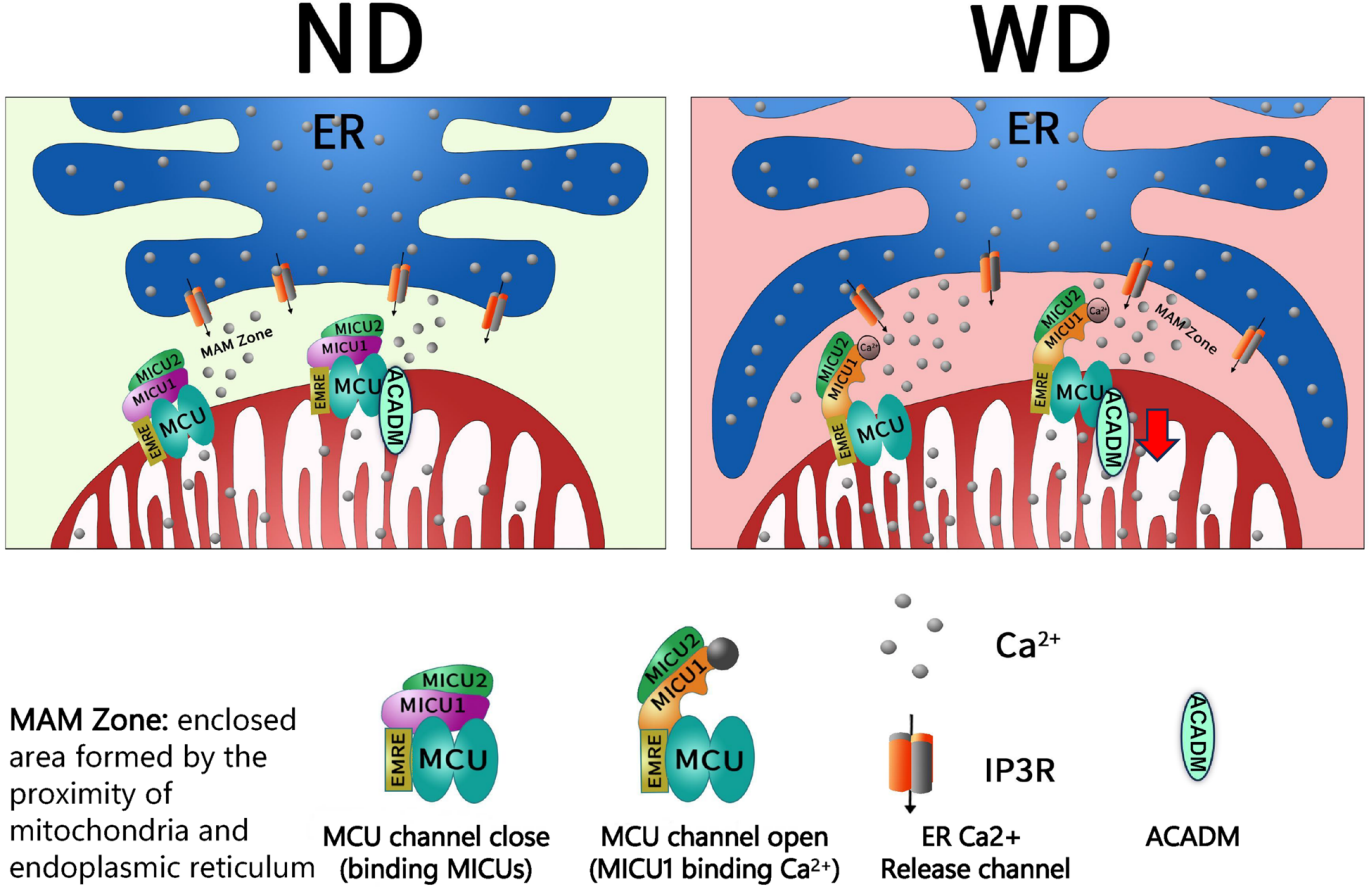
WD induces mitochondrial calcium overload graphical abstracts ND mean normal diet; WD mean west diet; ER mean Endoplasmic reticulum; In atherosclerosis, a west diet induces endoplasmic reticulum-mitochondrial connections, increasing the number of MAM. Ca^2+^ released from the ER bind to MICU1, thereby inactivating its role in closing the MCU channel. This induces mitochondrial calcium overload, reduces ACADM expression, and promotes lipid deposition.

In summary, a high-fat, high-cholesterol diet induced ER contact with mitochondria, leading to increased formation of MAM. Calcium ions released from the ER result in elevation of calcium concentration in the mitochondria. The excess calcium ions bind to MICU1, inhibiting its function as a blocker of MCUs and causing the MCUs to remain open. This leads to mitochondrial calcium overload, reduces ACADM expression, and induces mitochondrial oxidative stress, thereby promoting lipid deposition (Fig. 8).

## Discussion

In the present study, we found that cholesterol promotes lipid deposition in VSMCs while inducing mitochondrial calcium overload. The use of MCU inhibitors alleviates cholesterol-induced mitochondrial calcium overload and reduces lipid deposition. Further studies confirmed that cholesterol promotes the formation of MAMs, mimics MAMs to promote lipid accumulation and mitochondrial calcium overload, while inhibiting MAMs alleviates lipid accumulation and calcium overload. We confirm that extra-mitochondrial calcium ions resulting from increased MAMs bind to MICU1, leaving the latter in a runaway state and unable to close MCU channels, which in turn leads to mitochondrial calcium overload. Upon mitochondrial calcium overload, it may damage mitochondria by decreasing ATP synthesis and promoting ROS release in VSMCs, which in turn dysfunctions them and promotes lipid deposition.

Mitochondrial calcium homeostasis is strongly associated with a variety of diseases. Mitochondrial calcium flux has been shown to be associated with Cardiac Ischemia–Reperfusion Injury and Heart Failure [16], Stroke [17], Neurodegenerative Disease [18], Alzheimer’s disease [19], Parkinson’s disease [20], Amyotrophic lateral sclerosis [21], Diabetes and Obesity [22]. The research mainly focuses on cardiovascular and nervous system diseases. Sun et al. reported that endothelial MICU1 prevents vascular inflammation and atherosclerosis by maintaining mitochondrial homeostasis [23]. This study focuses on how MICU1 regulates SIRT1 expression to influence atherosclerosis. Its significance is substantial, yet research on its regulation of mitochondrial calcium homeostasis remains limited. Our study confirmed that mitochondrial calcium overload of VSMCs in atherosclerosis, and regulation of mitochondrial calcium can alleviate atherosclerosis.

MAMs are relatively confined regions of mitochondria and ER that are tightly connected to each other. MAMs plays a pivotal regulatory role in multiple cellular activities, such as Ca^2+^ and lipid transfer from the ER to mitochondria [24, 25]. In this study, increases in MAM were observed in cholesterol-treated smooth muscle cells, suggesting that MAM may play an unknown role during atherosclerosis (Fig. 5b-d). Then we modeled the formation of MAM using the ER-Mito linker gene expression vector, which expresses proteins that would target the outer mitochondrial membrane (OMM) at the N-terminus and the endoplasmic reticulum at the C-terminus (Fig. 5e). It was demonstrated that increasing MAMs stimulates mitochondrial calcium overload and increases lipid deposition.

Studies in Fig. 6 confirmed that MICU1 acts as a key regulator of mitochondrial calcium homeostasis, but its regulation of mitochondrial calcium levels is not positively correlated with lipid deposition (Fig. 6g-j). This is inconsistent with our speculation that mitochondrial calcium overload promotes lipid deposition and that lowering mitochondrial calcium alleviates lipid accumulation. After consideration, we found the only variable for both experiments-cholesterol. After the study, it was found that cholesterol invalidated the effect of MICU1 in clogging the MCUs (Fig. 7h&i). Mutation of MICU1 revealed that reducing the sensitivity of MICU1 to calcium ion binding allows MICU1 to function as a blocker of MCUs, which in turn alleviates mitochondrial calcium overload (Fig. 7g-k). The final conclusion remains that mitochondrial calcium overload promotes lipid deposition and inhibition of mitochondrial calcium overload alleviates lipid accumulation.

This study also has limitations. At present, research on mitochondrial calcium homeostasis mainly focuses on in vitro studies, and this study is no exception. The reason for this phenomenon is that there is not enough cutting-edge technology to detect changes in mitochondrial calcium levels in vivo. Moreover, this study did not design mitochondrial calcium homeostasis related gene edited mice, which can be used to confirm our conclusion. Furthermore, all mice used in this study were male; female animals also warrant subsequent investigation. These are the research direction we are currently and will be conducting in the future.

At present, researchers have developed many drugs to treat diseases induced by mitochondrial calcium homeostasis imbalance [26]. Arduino et al. identified mitoxantrone as a direct and selective inhibitor of MCU from more than 600 clinical used drugs [27]. The oxygen-bridged dinuclear ruthenium amine complex Ru360 is considered a widely used MCU inhibitor [28]. Therefore, these two inhibitors were used in this study and the data demonstrated that they could alleviate WD-induced imbalance of mitochondrial calcium homeostasis and Atherosclerosis. At present, there is no specific drug for atherosclerosis. The application of MCU inhibitors may provide research directions for subsequent drug development.

## Materials and methods

### Animals

Five-week-old male ApoE^−/−^ mice were purchased from GemPharmatech Co., Ltd (Nanjing, China) and kept in individual cages on a 12-h light/dark cycle with free access to water and food at room temperature. At week 8, divided into four groups (*n* = 5). (1) CN, normal diet with injected saline; (2) WD, western diet with injected saline; (3) WD + Ru360, western diet with injected Ru360; (4) WD + Mitox western diet with injected mitoxantrone. The mice were fed with a Normal Diet (ND) (AIN93) or Western Diet (WD) for 12 weeks. Mice were tested for body weight every 2 weeks, and drug injections were given every 5 days for 16 weeks. At the end of the experiment, the mice were anesthetized by inhalation of 3% isoflurane, bled by cardiac puncture, and euthanized by an anesthetic overdose with isoflurane, and the entire aorta and blood were harvested for further analysis.

### Human disease data

The human data of atherosclerosis were taken from the Gene Expression Omnibus, Accession Number GSE226790.

### Cell culture

Mouse VSMCs were cultured in DMEM medium (C11885500BT, Gibco) supplemented with fetal bovine serum (SA201.02, CellMax.

, China) and 1% penicillin–streptomycin (SC118-01, Seven, China). Rat vascular smooth muscle cells (R-VSMCs) were extracted from rat aorta. The R-VSMC cell culture medium was DMEM (Gibco) containing 10% FBS and 1% penicillin–streptomycin. For simulating HFCD in vitro, 30 mg/kg cholesterol (C4951, Sigma-Aldrich, USA) were conjugated to DMEM medium.

### Triglyceride, total cholesterol content detection

Cells or tissues were prepared according to the instructions of the A110-1–1 Triglyceride Assay Kit, A111-1–1 Total Cholesterol Assay Kit (Nanjing Jiancheng Bioengineering Institute, China). The microplate reader (Tecan M200 PRO) recorded the contents of TG, TC, then normalized using the protein concentration [29].

### Pathological section and staining

After 24 h of cholesterol treatment on VSMCs, fix with 4% paraformaldehyde for 30 min, stain the cells with Oil Red O (G1260, Solarbio, Beijing, China) for 30 min, rinse with 50% isopropanol and wash twice with PBS.

Embed mouse aortas in optimal cutting temperature compound. Create a frozen cross-Sect. (4 μm thick) and dry at room temperature for 15–20 min. Soak in isopropanol for 3–5 min, stain with Oil Red O for 10 min, wash with 60% isopropanol solution for 3 min, rinse with distilled water, counterstain the cell nucleus with hematoxylin for 5 min, differentiate and return to blue, and seal with a sealing agent.

#### HE

Paraffin sections were immersed in xylene three times, 10 min each time, followed by absolute ethano three times, 5 min each time. Hematoxylin staining for 2 min, 1% hydrochloric acid alcohol differentiation for 3 s, eosin staining solution for 30 s, washing with running water, dehydrated sealing, microscopic examination.

#### Masson

Paraffin sections were stained with hematoxylin for 5 min. After washing for 3 min, Masson ponceau acid complex red fluid was added for 5 min. Place the sections in phosphomolybdic acid for differentiation for a few seconds and then wash with water. Stain in aniline blue stain for 5 min and then rinse with 0.2% glacial acetic acid. dehydrated, permeabilized and sealed in neutral resin, microscopic examination [30].

### Calcium staining

Fluorometric measurements of [Ca^2+^] cytosolic were performed as described [31]. Briefly, saponin-permeabilized cells were resuspended in 1.5 ml of intracellular medium containing 120 mM KCl, 10 mM NaCl, 1 mM KH_2_PO_4_, 20 mM Tris-HEPES at pH 7.2, and supplemented with protease inhibitors, 2 mM MgATP, 2 μM Tg and maintained in a stirred thermostated cuvette at 36 °C. The extramitochondrial Ca^2+^ concentration was assessed using the ratiometric Ca^2+^ probe fura2/FA. Fura fluorescence was recorded using 340–380 nm excitation and 510 nm emission. Calibration of the fura signal was carried out at the end of each measurement, adding 1 mM CaCl_2_, followed by 10 mM EGTA/Tris, pH 8.5.

To measure mitochondrial, ER, and cytosolic calcium, permeabilized cells were loaded with Rhod-2 AM (5 µM), Fluo-5N (5 µM), and Fluo-4 AM (5 µM), respectively. The fluorescence levels of Fluo-4 AM, Fluo-5N and Rhod-2AM were determined at 494/516 nm (Ex/Em), 494/516 nm (Ex/Em) and 552/581 nm (Ex/Em) respectively To further validate this result, Ca^2+^ in mitochondria were assayed using pCMV-CEPIA2mt fluorescent vectors which is specifically expressed in mitochondria and produces green fluorescence when bound to Ca^2+^ [15]. Data are presented as F/F0.

### Western blot, co-immunoprecipitation, immunofluorescence staining

RIPA lysis buffer containing 1 mM PMSF was used to lyse cells. The total protein concentration was determined using a BCA protein assay kit. After separation via 10% SDS-PAGE, the proteins were transferred to polyvinylidene fluoride (PVDF) membranes and blocked with 5% nonfat dry milk in Tris-buffered saline containing Tween-20 for 1 h at room temperature. Subsequently, the PVDF membranes were incubated with the antibodies Primary antibody incubation was performed overnight at 4 °C, followed by incubation with the appropriate secondary antibody for 1 h at room temperature. The protein expression was detected using an ECL detection kit (HY-K1005, MedChemExpress, USA) and normalized using the β-actin levels [32]. For co-immunoprecipitation, A/G magnetic beads were used to incubate with antibodies, and relevant proteins were pulled down then western blot was performed. Primary antibodies MCU (67,735–1, Proteintech, dilution rates 1:2000), MICU1 (12,524, CST, dilution rates 1:2000), β-actin (66,009–1, Proteintech, dilution rates 1:1000), ACADM (sc-365448, Santa Cruz, dilution rates 1:1000) primary antibodies was used to perform experiments.

Paraffin sections were preincubated with 10% goat serum for 30 min and then incubated with anti-α-SMA (67,735–1, Proteintech) primary antibodies. Secondary antibodies were goat anti-mouse 594 (SA00013-3, Proteintech). Slices were sealed using a DAPI-containing sealer (H0621-V341, SouthernBiotech). Images were captured by a Confocal microscope (DM6000 CFS, Leica).

For immunofluorescence staining of mouse VSMC, cells were washed with PBS, fixed with 4% paraformaldehyde for 15 min at room temperature, and sealed with 10% goat serum for 30 min. Then cells were incubated overnight at 4 °C with anti-Calnexin (66,903–1, Proteintech, dilution rates 1:100), anti-MCU (26,312–1-AP, Proteintech, dilution rates 1:50). After PBS washing, fluorescein-labeled secondary antibodies were incubated for 1 h at room temperature. Images of the cells were captured using Confocal microscope [33].

### RT-qPCR

Total RNA was isolated with a total RNA extraction kit (R6934-01, OMEGA, USA). After RNA concentration was determined using a microplate reader. An equal amount of RNA for each sample was reverse transcribed to cDNA using PCR conditions of 95 °C for 3 min, followed by 40 cycles of 95 °C for 10 s, 60 °C for 1 min, and 72 °C for 10 s. Cycle threshold values were collected and normalized to that of housekeeping gene, and the fold change in gene expression was calculated with the △△Ct method. Primer sequence of qPCR is: *18S* forward: gtaacccgttgaaccccatt, reverse: ccatccaatcggtagtagcg. *MCU* forward: tcgtggagaggttagaggac, reverse: agaatgccaaactgggtgg. *MICU1* forward: cttgaatggagacggagagg, reverse: acttgagggtgttcccagtg. *HMGCR* forward: ttcagacgggtgttgcatcac, reverse: tgagcgtgaacaagaagaaccag. *ABCA1* forward: ggcaacaaacgaaagctc, reverse: cttagggcacaattcca*. TSPO* forward: gagcctactttgtacgtggcga, reverse: gctctttccagactatgtaggag*. STARD1* forward: atgcggtccacaagttcttc, reverse: *aaggctggaagaaggaaagc. CYP27A1* forward: acttgccctcctgtctcatc, reverse: ctatgtgctgcacttgccc. *PACS2* forward: gctccaaacgaatcctgcg, reverse: ttcagacgggtgttgcatcac.

### Lipidomics analysis

Lipidomics analysis was completed by the Shanghai applied protein technology Bio Institute [34]. The frozen sample was crushed using a mixer mill with a zirconia bead for 1.5 min at 30 Hz. The resulting powder was weighed and 100 mg was extracted overnight with 1.2 ml 70% aqueous methanol at 4 °C. Following centrifugation at 12,000 rpm for 10 min, the extracts were filtered before UPLC-MS/MS analysis. The sample extracts were analyzed using an UPLC-ESI–MS/MS system (UPLC, Shim-pack UFLC SHIMADZU CBM30A system; MS, Applied Biosystems 4500 Q TRAP). LIT and triple quadrupole (QQQ) scans were acquired on a triple quadrupole-linear ion trap mass spectrometer (Q-TRAP), API 4500 Q-TRAP UPLC/MS/MS System, equipped with an ESI Turbo Ion-Spray interface, operating in positive and negative ion mode and controlled by Analyst 1.6.3 software (AB Sciex). The criteria for determining differential metabolites were: Fold change ≥ 2 or ≤ 0.5, and Variable Importance in Projection (VIP) value ≥ 1.

### CPT1 activity detection

CPT1 enzymatic activity was detected by measuring the release of CoA-SH from palmitoyl-CoA using the general thiol reagent DTNB [35]. The DTNB buffer (116 mM Tris–HCl pH 8.0, 0.09% Triton X-100, 1.1 mM EDTA, 0.12 mM DNTB) and cell lysate were mixed and incubated at room temperature for 30 min. The absorbance of buffer at 405 nm was defined as background. Then 100 μM palmitoyl-CoA and 5 mM L-carnitine were added to the mixture. After a 20 min incubation at 37 °C, the absorbance was measured at 405 nm. The difference between readings with and without substrates was normalized to the Bradford protein concentration. CPT1 enzymatic activity was defined as millimoles of CoA-SH released per milligram of protein.

### Proximity Ligation Assay (PLA)

Proximity ligation assay based Duolink® In Situ Red Starter Kit Mouse/Rabbit (DUO92101, Sigma). First, fix and permeabilize cells cultured on the slide, add 1 drop (~ 40 µL) of Blocking Solution to each sample. Dilute primary antibody to suitable concentration (consistent with immunofluorescence) in the Antibody Diluent and incubate the samples. Then dilute the PLUS and MINUS PLA probes 1:5 in the Antibody Diluent and incubate the samples for 1 h. Dilute the 5 × Ligation buffer 1:5 in high purity water and mix and incubate the samples for 30 min. Dilute the 5 × Amplification buffer 1:5 in high purity water and mix and incubate the samples for 100 min. Observe images under a confocal microscope and perform quantitative analysis using Image J.

### ATP and ROS detection

Cellular ATP content was measured using a luciferin/luciferase-based kit (S0026, Beyotime Biotechnology, China). Briefly, a standard curve was plotted using ATP standards with known concentrations. Each 6-well plate was loaded with 200 μL of lysis buffer. After centrifugation at 4 °C, the supernatant was removed for detection. The Luminometer method was used to calculate ATP content by referencing the standard curve. The dihydroethidium (DHE) staining for ROS detection (S0033S, Beyotime Biotech, China) was utilized to detect intracellular ROS. In brief, cells were incubated with the fluorescent probe DHE for 30 min at 37 °C in the dark. Fluorescence intensity was detected using a fluorescence microscope (OLYMPUS, BX53F2) to assess ROS levels.

### Oxygen consumption rate

Oxygen consumption rate was measured by extracellular flux (XF24) analyzer (Seahorse Bioscience). cells were seeded at 2 × 10^4^ cells per well in 24-well plates. The probe plate was hydrated with the calibration solution (pH 7.4) and a 2 mM glutamine assay solution (pH 7.35 ± 0.05) was prepared. The probe plate was hydrated with the calibration solution (pH 7.4) and a 2 mM glutamine assay solution (pH 7.35 ± 0.05) was prepared, and incubated for 16–20 h at 37 °C and 5% CO2. The oxygen consumption rate drugs concentrations were as follows: oligomycin (final concentration 1 μM), carbonyl cyanide p-trifluoro-methoxyphenyl hydrazone (FCCP; final concentration 1 μM) and rotenone/antimycin A (ROT/AA) (final concentration 0.5 μM) [36].

### Transmission electron microscope observation

Cells were treated for 24 h and washed twice with PBS. Fresh culture medium was added to scrape off the cells and centrifuge them into clusters. The supernatant was discarded and fixed with 2.5% glutaraldehyde fixative along the centrifuge tube wall for 5 min. After centrifugation, the supernatant was removed and 1 ml of 2.5% glutaraldehyde fixative was added at 4 ℃. Electron microscopy sectioning and observation were completed on the Core Facilities and Centers of Hebei Medical University.

### Statistics and analysis

All experiments were repeated at least in triplicate, and statistical analysis was done using independent values. All data are presented as mean ± SD. Statistical analysis was performed using Student’s unpaired two-tailed t test (for two groups) or analysis of variance (ANOVA) (for multiple groups). One-way ANOVA with Tukey’s test was conducted for comparisons. The differences were considered significant at *p* < 0.05 (*). Values with different letters were significantly different.

## Supplementary Information

Supplementary Material 1

## Data Availability

The data that support the findings of this study are available from the corresponding author upon reasonable request.

## Abbreviations

VSMCs: Vascular smooth muscle cells
MAMs: Mitochondria-associated membranes
MCU: Mitochondrial calcium uniporter
MICU1: Mitochondrial calcium uptake 1
ER: Endoplasmic reticulum
LDLs: Low-density lipoproteins
oxLDL: Oxidized LDLs
ROS: Reactive oxygen species
mtDNA: Mitochondrial DNA
TG: Triglyceride
TC: Total Cholesterol
ND: Normal diet
WD: Western diet
