# Supplementary material for: Cholesterol induced-mitochondrial calcium dysregulation facilitates atherosclerosis by promoting lipid accumulation in vascular smooth muscle cells
Source: Mol Biomed. 2025 Dec 2;6:129. doi: 10.1186/s43556-025-00384-2 (PMC12672999; doi:10.1186/s43556-025-00384-2)
Supplement: Supplementary file 1 — Supplementary Material 1 [file 43556_2025_384_MOESM1_ESM.doc]

**Supplementary materials**

**Title:** Cholesterol induced-mitochondrial calcium dysregulation facilitates atherosclerosis by promoting lipid accumulation in vascular smooth muscle cells

**Authors:** Zhiwang Zhang^1^, Fan Yang^2^, Wei Wang^1^, Qi Cao^1^, Long Zhang^1^, Yu Zhang^1^, Dong Ma^1^, Xinhua Zhang^1^, Jinkun Wen*^1^, Bin Zheng*^1，3^

**Author affiliations:** 1. Department of Biochemistry and Molecular Biology, Key Laboratory of Neural and Vascular Biology, Ministry of Education, Hebei Medical University, Shijiazhuang, 050017, China; 2. College of Integrative Medicine, Hebei University of Chinese Medicine, Shijiazhuang, 050200, China. 3.Hebei Key Laboratory of Cardiovascular Homeostasis and Aging, Hebei Medical University, Shijiazhuang, 050017, China

***Corresponding author:** Prof. Bin Zheng, E-mail: doublezb@hebmu.edu.cn; Jin-kun Wen, E-mail: wjk@hebmu.edu.cn. Department of Biochemistry and Molecular Biology, Key Laboratory of Neural and Vascular Biology, Ministry of Education, Hebei Medical University, Shijiazhuang, 050017, China.


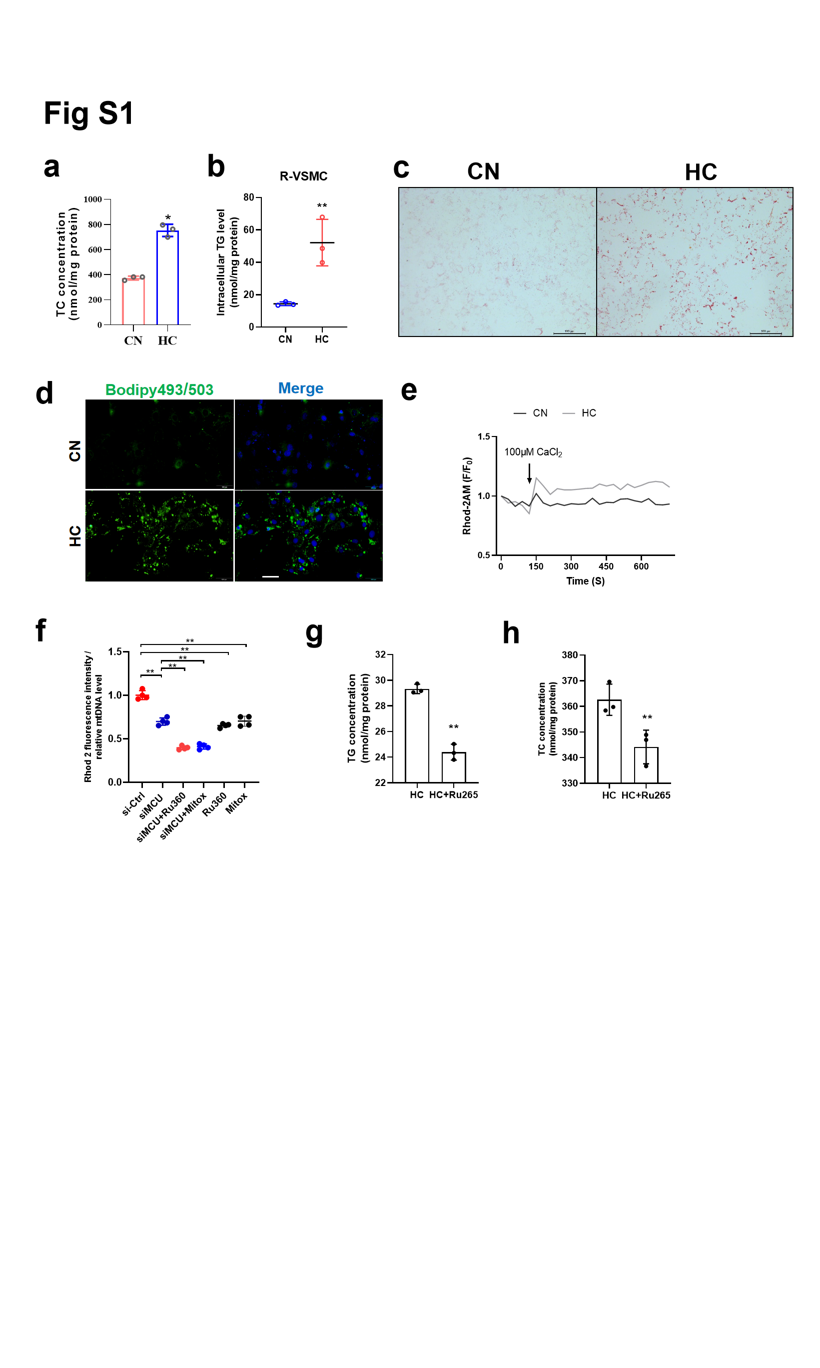


Figure S1

(a) Total cholesterol content. (b) Triglyceride content (Rat-VSMC). (c) Oil red O staining of lipid droplets bar=100μm. (d) Bodipy staining of lipid droplets. bar=200μm (e) Mitochondrial calcium change curve(F/F0). (f) Mitochondrial calcium level. (g) Triglyceride content. (h) Total cholesterol content.


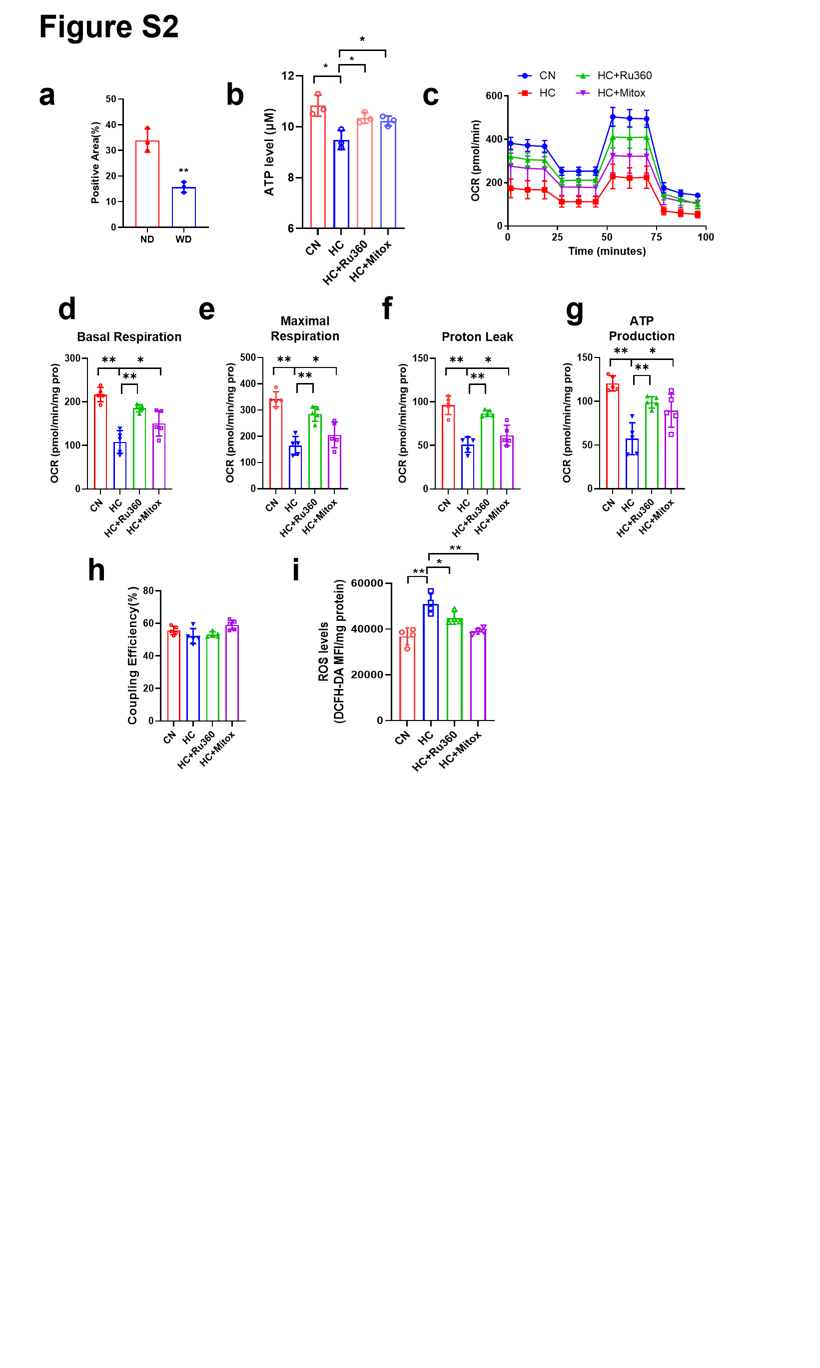


Figure S2

(a) Immunohistochemical positive area. (b) ATP level. (c) OCR curve. (d) Basal respiration. (e) Maximal respiration. (f) Proton leak. (g) ATP production. (h) Coupling efficiency. (i) ROS levels.


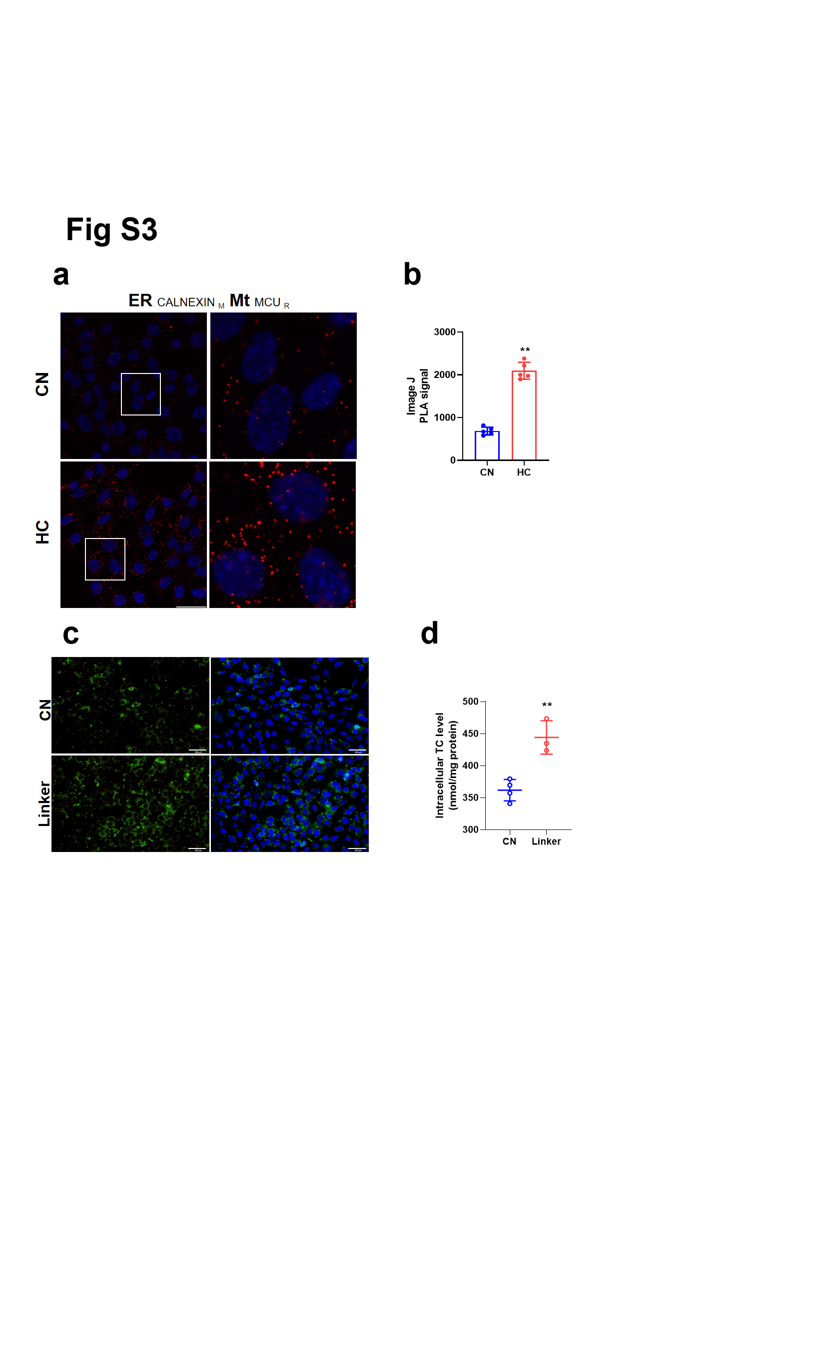


Figure S3

(a) Proximity ligation assay, antibody Calnexin (mouse); antibody MCU (rabbit). (b) Image J PLA signal. (c) Bodipy staining of lipid droplets bar=200μm. (d) Total cholesterol content.


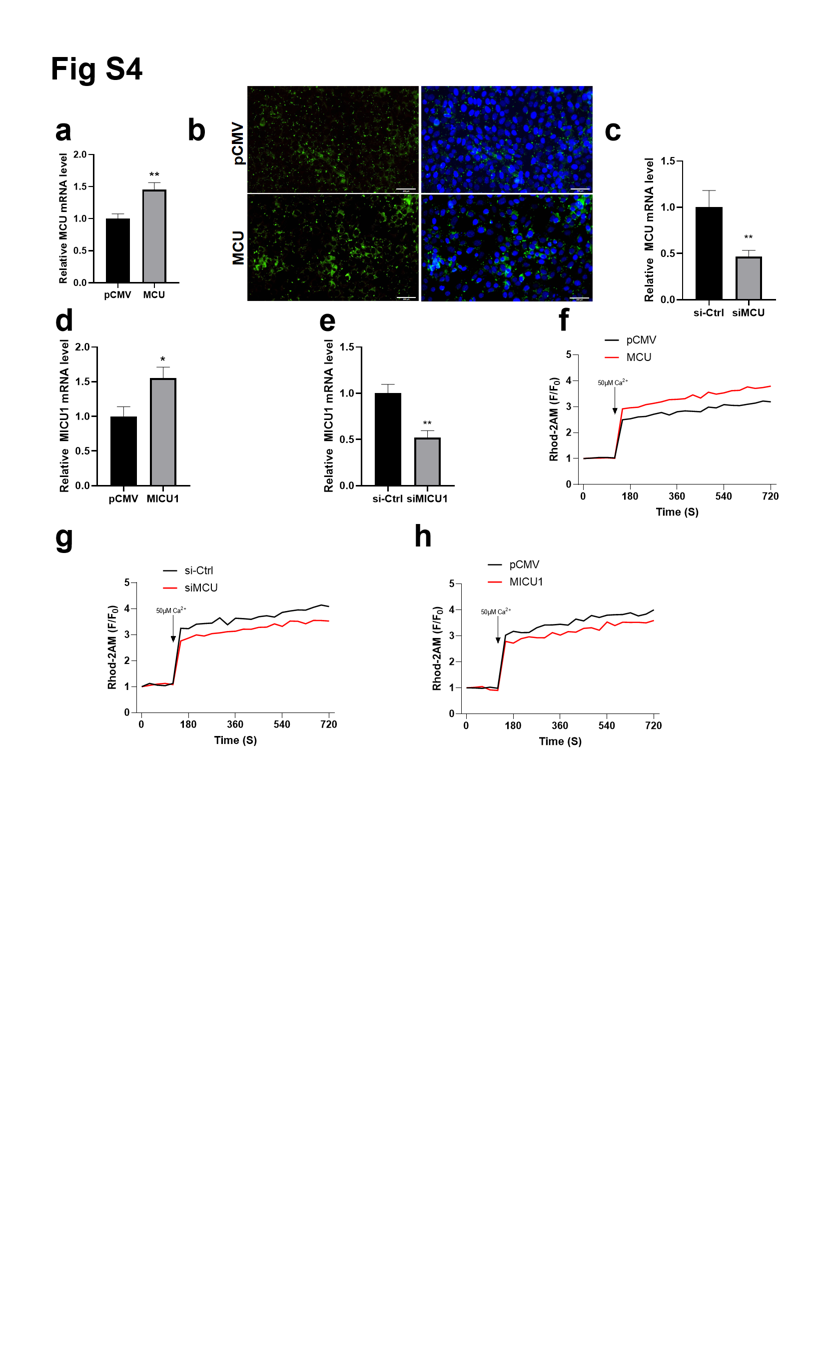


Figure S4

(a) Mcu mRNA expression level. (b) Bodipy staining of lipid droplets bar=200μm. (c) Mcu mRNA expression level. (d)(e) Micu1 mRNA expression level. (f) Mitochondrial calcium change curve(F/F0). (g) Mitochondrial calcium change curve(F/F0). (h) Mitochondrial calcium change curve(F/F0).
